# Editorial: Robotic grasping and manipulation of deformable objects

**DOI:** 10.3389/frobt.2022.1108038

**Published:** 2023-01-06

**Authors:** Joao Bimbo, Minas Liarokapis, Monica Malvezzi, Gionata Salvietti

**Affiliations:** ^1^ Universidade Lusófona, Lisbon, Portugal; ^2^ Department of Mechanical and Mechatronics Engineering, The University of Auckland, Auckland, New Zealand; ^3^ Department of Information Engineering and Mathematics, University of Siena, Siena, Italy

**Keywords:** soft robotic grippers, robotic grippers, grasping, manipulation, grasp modelling, grasp control, modeling deformable objects

In most robotics applications the physical interaction between the robot and the environment is a fundamental feature. In this context, the ability to grasp and manipulate objects with robotic systems and devices in a safe and robust way has been an important and challenging Research Topic in robotics for at least five decades. This ability is particularly important when high performance is required and/or when complex tasks need to be executed in a dynamic and unstructured environment.

The problem has been traditionally modeled quasi-statically by considering ideal conditions that allow its representation as a standard multibody system, in which both the manipulator links and the object are rigid bodies, robot joints are ideal, contacts are defined as points, friction can be described using simple models, etc. All these assumptions allowed managing the problem with simple mathematical models that can be used in the design of robotic devices, as well as in the development. Of the required sensing system and control strategies for the execution of complex tasks.

Nowadays, robots are increasingly spreading outside of rigid and structured industrial production cells to dynamically changing environments which may include humans, opening a wide set of new applications and challenges, in which the objects manipulated by the robots (e.g., fruits and vegetables, fabric, etc.) and even the robotic structures themselves are made of compliant materials, have uncertain shapes and positions, and can undergo large, complex deformations when a force is applied to them. In these cases, the clear and well-defined modeling assumptions mentioned earlier cannot be applied.

Robots are required to interact with deformable objects in several industrial and production environments, for instance, manufacturing, logistics, agriculture, as well as interactive robotic applications, such as robotic surgery, household robotics and rehabilitation robotics, to name a few.

In this Research Topic, we focus on the interesting and still open research challenge of grasping and manipulating deformable objects.

The problems include: 1) modeling and estimation of a manipulated object’s shape when deformed, 2) estimation of an object’s material properties, such as elasticity and plasticity, 3) object tracking and state estimation during manipulation,4) gripper design that exploit softness and adaptability to avoid object or robot damage, 5) robot perception and sensor systems for grasping and manipulation with deformable systems, 6) manipulation planning and control for deformable systems, 7) learning and adaptation for deformable object grasping and manipulation, etc. It is clear that addressing these challenges involves almost all aspects of robotics: modeling and simulation, hardware design, sensing, planning, and control, etc.

The papers included in this Research Topic show how the theme of robotic grasping and manipulation of deformable objects can be tackled from different and complementary perspectives, from the design of suitable grippers and end effectors that exploit softness and are therefore capable of adapting to grasped objects in a robust and safe/delicate manner, to the development of sensing systems and the planning and control schemes of these deformable robotic devices.

Classical grasp and manipulation models are based on the hypothesis that the object is a rigid body, but how should this hypothesis be modified to include deformable objects? Concerning the modeling aspect (Arriola-Rios et al.), provides an overview on the available methodologies for representing the shape and dynamics of deformable objects. From this starting point, the authors propose a review of existing work on how such models can be learned and estimated and how motion planning and control methods can be used to achieve desired deformations.

The great number of Degrees of Freedom of deformable objects and the complexity of the relationships between their configuration (shape) and the applied forces requires the development of specific methodologies for estimating and controlling the deformation. In (Sanchez et al.) the authors present a novel pipeline to solve this problem based only on force sensing and FEM (Finite Element Modelling) of the object. The pipeline combines a sensor model, a deformation model and a pose controller. The sensor model calculate the contact forces for the deformation model that then updates the volumetric mesh of the manipulated object. The controller is used to deform the object so as for a mesh pose to reach a desired goal pose.

An interesting application is presented in (Aghajanzadeh et al.) where the general problem of deformable linear object manipulation is investigated. More specifically, the authors consider an elastic linear object where one of its endpoints is fixed, and another point can be grasped by a robotic arm. For this structure, different mathematical models relating the applied grasping forces to object configuration are available. The authors propose a model-free method to control the state of an arbitrary point on the object, allowing the robot to manipulate the object without any knowledge of the model parameters or offline information of the object’s deformation, by using an adaptive control strategy. The main application considered in the paper is plant grasping in agriculture.

The availability of a benchmark and a testing procedure is important to assess the performance of grasping and manipulation systems and compare them with other available solutions. In this context, the National Institute of Standards and Technology is developing performance tests and associated artifacts to benchmark research in the area of robotic assembly, including many non-rigid component operations representative of wire harness and belt drive assemblies to support research in the area of grasping and manipulation of deformable objects. The paper (Kimble et al.) presents a set of four task boards representing benchmarks for grasping evaluation, along with scoring metrics and methods to compare robot system assembly times with human performance.

